# Embryonic size and growth and adverse birth outcomes: the Rotterdam Periconception Cohort

**DOI:** 10.1093/humrep/deae212

**Published:** 2024-09-17

**Authors:** J A Roelants, M J Vermeulen, S P Willemsen, J V Been, A H Koning, A J Eggink, K F M Joosten, I K M Reiss, R P M Steegers-Theunissen

**Affiliations:** Department of Neonatal and Pediatric Intensive Care, Division of Neonatology, Erasmus MC—Sophia Children’s Hospital, Rotterdam, The Netherlands; Department of Obstetrics and Gynecology, Erasmus MC, University Medical Center Rotterdam, Rotterdam, The Netherlands; Department of Neonatal and Pediatric Intensive Care, Division of Neonatology, Erasmus MC—Sophia Children’s Hospital, Rotterdam, The Netherlands; Department of Obstetrics and Gynecology, Erasmus MC, University Medical Center Rotterdam, Rotterdam, The Netherlands; Department of Epidemiology, Erasmus MC, University Medical Center Rotterdam, Rotterdam, The Netherlands; Department of Neonatal and Pediatric Intensive Care, Division of Neonatology, Erasmus MC—Sophia Children’s Hospital, Rotterdam, The Netherlands; Department of Obstetrics and Gynecology, Erasmus MC, University Medical Center Rotterdam, Rotterdam, The Netherlands; Department of Public Health, Erasmus MC, University Medical Center Rotterdam, Rotterdam, The Netherlands; Department of Obstetrics and Gynecology, Erasmus MC, University Medical Center Rotterdam, Rotterdam, The Netherlands; Department of Pathology, Erasmus MC, University Medical Center Rotterdam, Rotterdam, The Netherlands; Department of Obstetrics and Gynecology, Erasmus MC, University Medical Center Rotterdam, Rotterdam, The Netherlands; Department of Neonatal and Pediatric Intensive Care, Division of Pediatric Intensive Care, Erasmus MC-Sophia Children’s Hospital, Rotterdam, The Netherlands; Department of Neonatal and Pediatric Intensive Care, Division of Neonatology, Erasmus MC—Sophia Children’s Hospital, Rotterdam, The Netherlands; Department of Neonatal and Pediatric Intensive Care, Division of Neonatology, Erasmus MC—Sophia Children’s Hospital, Rotterdam, The Netherlands; Department of Obstetrics and Gynecology, Erasmus MC, University Medical Center Rotterdam, Rotterdam, The Netherlands

**Keywords:** first trimester, DOHaD, pregnancy, embryonic volume, life course, 3D ultrasound

## Abstract

**STUDY QUESTION:**

Is early embryonic size and growth in the first trimester of pregnancy associated with adverse birth outcomes?

**SUMMARY ANSWER:**

Larger embryonic crown–rump length (CRL) and embryonic volume (EV) are associated with lower odds of adverse birth outcomes, especially small for gestational age (SGA).

**WHAT IS ALREADY KNOWN:**

Preterm birth, SGA, and congenital anomalies are the most prevalent adverse birth outcomes with lifelong health consequences as well as high medical and societal costs. In the late first and second trimesters of pregnancy, fetuses at risk for adverse birth outcomes can be identified using 2-dimensional ultrasonography (US).

**STUDY DESIGN, SIZE, DURATION:**

Between 2009 and 2018, singleton pregnancies were enrolled in this ongoing prospective Rotterdam Periconception Cohort.

**PARTICIPANTS/MATERIALS, SETTING, METHODS:**

This study included 918 pregnant women from a tertiary hospital in the Netherlands. Pregnancy dating was based on either a regular menstrual cycle (for natural pregnancies) or a conception date (for ART pregnancies). CRL and EV were measured using Virtual Reality software on 3-dimensional (3D) ultrasound scans, repeatedly performed around 7, 9, and 11 weeks of gestation. The main outcome measure was adverse birth outcome, defined as the composite of SGA (birth weight <10th percentile), preterm birth (<37th week of gestation), congenital anomalies (Eurocat criteria), stillbirth (>16th week of pregnancy), or early neonatal mortality (≤7 days of life). Reference curves for CRL and EV were constructed. Cross-sectional (CRL/EV <20th percentile at 7, 9, and 11 weeks of gestation) and longitudinal (CRL/EV growth trajectories between 6th and 13th weeks) regression analyses were performed, with adjustments for the participants’ educational level, smoking, parity, age, BMI, geographical background, mode of conception, and fetal sex.

**MAIN RESULTS AND THE ROLE OF CHANCE:**

Of the 918 pregnant women included, the median age was 32.3 years, and 404 (44%) pregnancies had been conceived via ART. In 199 (22%) pregnancies, there was an adverse birth outcome. Regression analyses showed that at 7 weeks of gestation onwards, embryos with a CRL <20th percentile had an ∼2-fold increased odds of adverse birth outcome (adjusted odds ratio (aOR) 2.03, 95% CI 1.21—3.39, *P* = 0.007). Similar associations were found for EV <20th percentile but were not statistically significant. These findings were mainly driven by the strong association between embryonic size and SGA (e.g. 7-week CRL: aOR 2.18 (1.16–4.09), *P* = 0.02; 9-week EV: aOR 2.09 (1.10—3.97, *P* = 0.02). Longitudinal growth trajectories of CRL, but not of EV, were associated with adverse birth outcomes. Both CRL and EV growth trajectories were associated with SGA.

**LIMITATIONS, REASONS FOR CAUTION:**

The tertiary hospital population and the availability of sophisticated 3D-ultrasound techniques limit the generalizability of this study to general populations and settings.

**WIDER IMPLICATIONS OF THE FINDINGS:**

Already very early in the first trimester of pregnancy, embryos with increased risks of an adverse birth outcome can be identified by using 3D-US and Virtual Reality. This expands the window of opportunity to enable the development of future interventions to potentially improve pregnancy outcomes and offspring health during their life-course.

**STUDY FUNDING/COMPETING INTEREST(S):**

This work was funded by the Department of Obstetrics and Gynecology, Erasmus MC, University Medical Centre, Rotterdam, The Netherlands. The authors declare no conflicts of interest.

**TRIAL REGISTRATION NUMBER:**

NL4115.

## Introduction

Preterm birth, small for gestational age (SGA) and congenital anomalies are the most common adverse birth outcomes worldwide and are associated with short- and long-term health consequences as well as high societal impact and costs (Kullinger *et al.*, 2016; Vos *et al.*, 2016). An early predictor for these adverse birth outcomes is impaired growth of the fetus. Previous studies using 2-dimensional (2D) ultrasonography (US) demonstrated that this relation can be identified from 10 weeks of gestation onward (Bukowski *et al.*, 2007; Mook-Kanamori *et al.*, 2010; Vafaei *et al.*, 2012; Simic *et al.*, 2017; Cho *et al.*, 2018; Patel *et al.*, 2022; Xu *et al.*, 2022). Although that may seem early in pregnancy, it may be too late for effective prevention or intervention. Current literature stresses the critical timing of the periconceptional period, starting 14 weeks before conception and ending around 10 weeks of gestation, for fetal programming and later health (according to the DOHaD (developmental origins of health and disease) paradigm) (Barker, 2007; Steegers-Theunissen *et al.*, 2016; Rousian *et al.*, 2021). Identification of embryos at risk before 10 weeks of gestation might therefore provide opportunities for timely interventions to improve short- and long-term health outcomes.

A major limitation of the commonly used 2D-US assessment of growth is that the smaller and younger the embryo, the more difficult it is to accurately measure embryonic size. New techniques, such as 3-dimensional (3D)-US and Virtual Reality (VR) software, improve embryonic visualization and provide an opportunity to assess volumetric measures (Rousian *et al.*, 2009, 2010; van Uitert *et al.*, 2013). These techniques can be used to adequately measure crown–rump length (CRL) and embryonic volume (EV) from 6 weeks of gestation onward (Rousian *et al.*, 2010; Verwoerd-Dikkeboom *et al.*, 2010). Whether these techniques help identify embryos at risk for adverse birth outcomes earlier is unknown.

To answer this question, we studied associations between CRL and EV measured using new 3D-US and VR techniques in the early embryonic period and later birth outcomes in pregnant women attending a tertiary hospital. We hypothesized that larger embryonic size and faster growth trajectories are associated with lower risks of adverse birth outcomes; in other words, there is a negative association between size/growth and adverse outcomes. We primarily explored this in a group of pregnant women with reliable estimations of pregnancy duration. Additionally, we also included pregnancies with less reliable pregnancy dating for external validity and generalizability to other research and clinical care.

## Materials and methods

### Study design

The Rotterdam Periconceptional Cohort (Predict Study) is an ongoing hospital-based prospective cohort of pregnant persons and their partners with 1-year follow-up after delivery, conducted at the department of Obstetrics and Gynecology of the Erasmus MC, University Medical Center, Rotterdam, The Netherlands (Steegers-Theunissen *et al.*, 2016; Rousian *et al.*, 2021). From this cohort, we selected singleton pregnancies enrolled between 2009 and 2018, with serial 3D-US scanning in the first trimester of pregnancy. Although of interest, we excluded pregnancies achieved after oocyte donation, miscarriages before the 16th week of gestation because of limited participant data, and those with complete missing information on birth outcome. In those who participated repeatedly in the Predict study, only the first pregnancy was used in the analyses. The STROBE guideline was used for this report.

### Ethical approval

The Central Committee on Research in The Hague and the local Medical Ethical Committee approved the study (MEC-2004-227). Written informed consent was obtained before enrollment from all participants and their partners.

### Study summary

A detailed study protocol of the Predict study has been published previously (Steegers-Theunissen *et al.*, 2016; Rousian *et al.*, 2021). In short, transvaginal 3D-US scanning was performed repeatedly between 6 and 13 weeks of gestation. In addition to this, participants filled in questionnaires during pregnancy and after delivery, with a focus on general characteristics, health conditions, and lifestyle.

### Ultrasound examinations

Transvaginal 3D-US scanning was performed repeatedly between 6 and 13 weeks of gestation using a 6–12 MHz transvaginal probe (General Electrics Voluson E8) combined with 4D View software (General Electrics Medical Systems, Zipf, Austria). Weekly 3D-US scans were performed in 2009–2013. For logistical reasons, the schedule was changed to scanning at 7, 9, and 11 weeks of gestation from 2013 onward (Steegers-Theunissen *et al.*, 2016). 3D-US was performed per protocol by trained and dedicated researchers.

The 3D datasets obtained were examined in the BARCO I-Space (Barco N.V., Kortrijk, Belgium). The BARCO I-Space is a four-walled CAVE^TM^-like VR system, allowing depth perception and 3D interaction with the projected scans (3D-VR) (Groenenberg *et al.*, 2005). For evaluation of embryonic size and growth, CRL (mm) and EV (cm^3^) were measured in the I-Space by trained researchers using the in-house developed V-Scope software. Reliability, technique, and methods of these measures have been described in detail previously, showing inter- and intra-observer agreement and intra-class correlation coefficients above 0.98 for both CRL and EV (Rousian *et al.*, 2009, 2010; Verwoerd-Dikkeboom *et al.*, 2010).

Missing ultrasound and measurement data had several inevitable reasons, including: (i) first measurements (7th week) being missing when study participation started after the 7th week of gestation (this was not uncommon in the Netherlands, as most patients have their first clinical visit after 8 weeks of gestation, and many participants were included after their first clinical visit); (ii) due date estimation being performed around 9 weeks of gestation, which sometimes, in retrospect, resulted in ultrasounds being performed outside the used timeframes (e.g. 7^+0^–7^+6^); (iii) technical and physical limitations, such as high BMI, interfering with the ability to adequately measure CRL and especially EV; and (iv) no-show or re-scheduling of the visit for any reason.

### Data collection

Data on participants’ health, lifestyle, pregnancy course, and birth outcome were obtained via questionnaires completed at enrollment, in mid-pregnancy, and shortly after delivery. Obstetrical and birth outcome data were cross-checked with the electronic medical files. Incomplete questionnaires were completed using electronic medical files. The following characteristics were obtained: age, geographical background, parity, mode of conception, periconceptional smoking, and folic acid supplement use. Periconceptional BMI was calculated from weight and length in the first questionnaire, or, if not available, from measurements at the first study visit.

The following data on birth outcome were obtained: date of delivery, sex, birth weight, gestational age (GA) at birth, birth weight percentiles, stillbirth, or early neonatal death (≤7 days of life), and congenital anomalies (“Eurocat criteria Guide 1.4 Section 3.3. Obtained on 06/09/2017,” n.d.). Birth weight percentiles adjusted for GA, parity, and sex were calculated using Dutch reference standards (Hoftiezer *et al.*, 2016, 2019).

### Pregnancy dating

Data on the mode of conception, the first day of the last menstrual period (LMP), as well as regularity and duration of the menstrual cycle were obtained in the first questionnaire and at the first research visit and then cross-checked with data in the electronic medical file. In naturally conceived pregnancies, the estimated date of delivery (EDD) was based on a regular menstrual cycle of 25–35 days and the first day of the LMP. If the duration of the menstrual cycle was 32–35 days, we adjusted for duration of the cycle according to the protocol (Steegers-Theunissen *et al.*, 2016). To detect potential recall bias, we checked correspondence between EDD based on LMP and that based on CRL measured on the 9-week 2D-US scan. If there was a difference of ≥7 days, an unknown date of the LMP, or an irregular menstrual cycle (<25 or >35 days), pregnancy dating was based on the 2D measurement of CRL around 9-weeks of gestation.

In pregnancies conceived via ART, including IVF with or without intracytoplasmic sperm injection, the EDD was calculated based on the date of oocyte retrieval. After cryo-embryo transfer, the EDD was calculated from the transfer day plus 19 days (Steegers-Theunissen *et al.*, 2016; Rousian *et al.*, 2021).

### Study groups

In total, we included 1241 pregnant women (full cohort), with pregnancy dating based on either LMP, CRL, or conception date. Previous studies showed that embryonic CRL growth is not uniform but shows variation according to individual genetic and environmental factors (Bottomley *et al.*, 2009; van Uitert *et al.*, 2013). This limits the reliability of dating based on CRL, which assumes uniform embryonic growth. Therefore, we focused our primary analyses on pregnancies with the most reliable EDD estimation (strictly dated), namely those dated on LMP and on conception date (ART pregnancies), and excluded pregnancies dated based on CRL. This led to a main study group of 918 pregnant women. For generalizability in relation to other studies and to clinical practice, in which CRL dating is standard of care, we performed sensitivity analyses for the full cohort, hence additionally including those with CRL-based pregnancy dating (N = 1241).

### Outcome measures

The main outcome measure was adverse birth outcome, defined as the composite outcome of SGA (birth weight below the 10th percentile), preterm birth (birth before 37 weeks of gestation), major congenital anomalies (based on EUROCAT Criteria), and perinatal mortality (stillbirth after 16 weeks of gestation or neonatal death within 7 days after birth). Secondary outcome measures explored separately included preterm birth, GA at birth, SGA, birth weight percentiles, and major congenital anomalies. Because of the expected low rate of stillbirth and neonatal death, these were not studied separately.

### Statistical analyses

Baseline characteristics are presented as mean (SD), median (interquartile range, IQR), or frequencies (n, %).

We assessed the association between CRL (mm) or EV (cm^3^) <20th percentile and birth outcome. This cut-off is based on the previously published report of Mook-Kanamori *et al.* (2010). To standardize the CRLs and EVs, we first developed reference curves for the (root) CRLs and (cube roots) EVs of our study population using the LMS method. These reference curves are used to estimate whether the CRL or EV falls below the 20th centile. After this, we performed cross-sectional analyses on associations between the standardized CRL and EV below the 20th percentile at 7 (range 7 0/7–7 6/7), 9 (9 0/7–9 6/7), and 11 (11 0/7–11 6/7) weeks of gestation and the outcome measures, using a (bias reduced) logistic regression model where the adverse birth outcome is the response (Kenne Pagui *et al.*, 2017).

All associations were explored in two models for all outcomes. The simple model (Model 1) adjusted only for GA at the moment of US. An extended model (Model 2) accounted for potential confounders, the selection of which was informed by previous studies in the same cohort and previous literature (Bottomley *et al.*, 2009; van Uitert *et al.*, 2013; Kullinger *et al.*, 2016). These were educational level (low/middle/high), smoking (yes/no), fetal sex (male/female), parity (nulliparous/multiparous), age, periconceptional BMI, geographical background (Western/non-Western), and mode of conception (natural/ART). Results presented in the manuscript are based on this extended model. The results are presented as (adjusted) odds ratios ((a)OR) with 95% CI.

To determine the additional value of longitudinal measurements compared to cross-sectional measurements, associations between longitudinal growth trajectories and adverse birth outcomes were studied. For this, we modeled individual CRL and EV growth trajectories between 6 and 13 weeks of gestation using linear mixed models. Approximate homoscedastic and normal distributions and linear associations of the growth parameters and GA were obtained by square root transformation of CRL and cubic root transformation of EV. The individual growth trajectories were summarized by the subject-specific random effects (intercept and slope), which were subsequently entered as covariates in a regression model. We report only the statistical significance of the association between growth and outcomes, as this complex statistical method does not yield interpretable effect estimates.

We used missing data imputation by chained equations to impute missing covariates by creating five data sets that were analyzed separately (van Buuren *et al.*, 2006). Results of these five analyses were then combined using Rubin’s Rules (Rubin, 2004). Given the hypothesis‐driven analysis and for ease of interpretation, multiple testing adjustment was not applied (Feise, 2002).

A two-tailed *P* value of <0.05 was considered statistically significant. Data were analyzed using SPSS (SPSS package 21.0, IBM, USA) and R (R: A language and Environment for Statistical Computing, version 3.4.1, R Core Team, Vienna, Austria).

## Results

Of 1557 pregnancies studied in the Predict Study, we included 1241 pregnancies (Supplementary Fig. S1). Of those, 918 were strictly dated (main study group) and in 323 participants, pregnancy dating was performed by CRL. The included and excluded pregnancies were largely comparable. Those excluded groups more often had missing information (Supplementary Table S1). A comparison based on the method of pregnancy dating showed that those with ART pregnancies were less likely to smoke and were more often primiparous (Supplementary Table S2).

### Main study group

The baseline characteristics of the main study group (n = 918) are presented in Table 1. The cohort consisted of participants who were mainly of Western origin, predominantly middle or highly educated, and most often nulliparous. Adverse birth outcomes occurred in 199 (22%) participants, mostly due to SGA (n = 110, 12%) and preterm birth (n = 73, 8%).

**Table 1.** Baseline characteristics.

		Main study group (N = 918)	Missing data
**Maternal characteristics**			
Age (years)		32.3 (29.3–35.6)	236
Geographical background	*Western*	770 (84%)	12
	*Non-western*	136 (15%)	
Educational level	*High*	510 (56%)	28
	*Middle*	310 (34%)	
	*Low*	70 (8%)	
Periconceptional BMI (kg/m^2^)		23.8 (21.4–26.7)	7
Folic acid use		904 (99%)	6
	*Preconceptional initation*	747 (81%)	25
Periconceptional smoking		142 (16%)	5
Nulliparous		540 (59%)	0
Conception mode	*Natural pregnancy*	514 (56%)	0
	*ART*	404 (44%)	
**Neonatal characteristics**			
Gestational age at birth (weeks^+days^)		39^+2^ (38^+1^–40^+2^)	0
	*Preterm birth*	73 (8%)	0
Birth weight (gram)		3340 (3000–3680)	0
Birth weight percentile		44 (22–71)	0
	*SGA*	110 (12%)	0
Sex	*Male*	451 (50%)	0
Major congenital anomaly		29 (3%)	32
Mortality	*Fetal*	11 (1%)	0
	*Early neonatal*	4 (1%)	0
Adverse birth outcome		199 (22%)	0

Data are presented in median (interquartile range) or number (percentages). SGA, small for gestational age.

**Figure 1.**
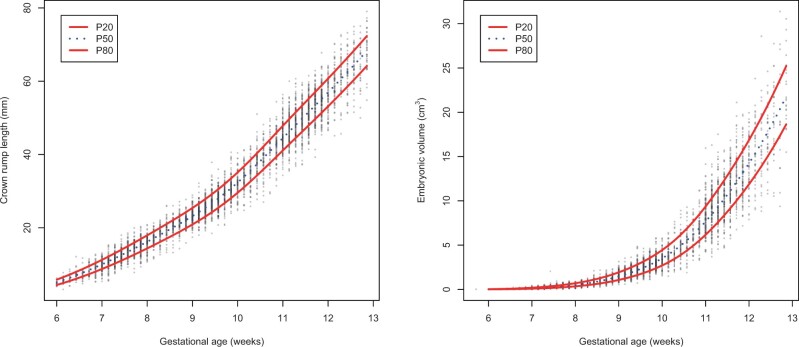
**Reference curves for CRL and EV.** Presented are crown–rump length (CRL) and embryonic volume (EV) reference curves. The red line represents the 20th and 80th percentiles, the blue dotted line represents the 50th percentile. On the *x*-axis is the gestational age (weeks) at ultrasound measurement, on the *y*-axis is the CRL (mm, left figure) and EV (cm^3^, right figure).

The median number of successful first-trimester 3D-US per pregnancy was three. CRL and EV were measured, respectively, in 481 and 414 (53%, 45%), 699 and 616 (76%, 67%), and 753 and 584 (82%, 64%) participants at 7, 9, and 11 weeks of gestation, respectively. In Fig. 1 the references curves for CRL and EV are presented. Mean CRL increased from 12.7 (SD 2.7, min–max 5.4–24.5) mm at 7 weeks to 49.8 (5.5, 46.0–53.6) mm at 11 weeks of gestation. Mean EV increased from 0.30 (SD 0.19, 0.16–0.40) cm^3^ at 7 weeks to 10.5 (3.0, 8.3–12.3) cm^3^ at 11 weeks of gestation.

### Primary analyses

#### Primary outcome

**Figure 2.**
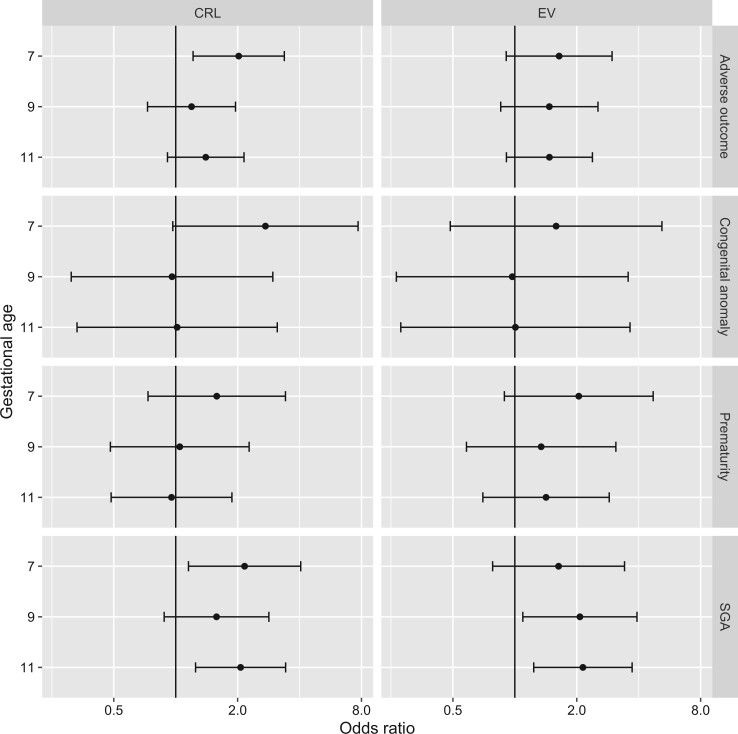
**Associations between embryonic size and birth outcomes in the main study cohort.** ORs are presented for CRL and EV <20th percentile (*x*-axis) for the different outcomes at the different time-points (*y*-axis). An OR >1 indicates an increased odds for the adverse outcome if embryonic size is <20th percentile. CRL, crown–rump length; EV, embryonic volume; SGA, small for gestational age; OR, odds ratio.

CRL <20th percentile was significantly associated with the composite outcome ‘adverse birth outcome’ at 7 weeks of gestation (Fig. 2). A pregnancy of an embryo with a CRL <20th percentile showed a higher odds of an adverse birth outcome: i.e. a 7-week CRL <20th percentile has a two times higher odd of an adverse birth outcome (adjusted odds ratio (aOR) 2.03 (95% CI 1.21—3.39), *P* = 0.007) (Fig. 2, Supplementary Table S3). For EV, none of the time-points were statistically significantly associated with adverse birth outcome. Embryonic growth, depicted by longitudinal CRL, but not longitudinal EV, was significantly associated with adverse birth outcome.

#### Secondary outcomes

CRL and EV <20th percentile were not associated with preterm birth separately (Fig. 2, Supplementary Table S3). However 7- and 11-week CRL and 9- and 11-week EV <20th percentile were associated with SGA (Fig. 2, Supplementary Table S3). At 11 weeks, CRL and EV below the 20th percentile were both associated with two times higher odds for SGA (CRL: aOR 2.10 (95% CI 1.27—3.50), *P* = 0.004; EV: aOR 2.17 (95% CI 1.25–3.87), *P* = 0.006). No associations were observed between CRL or EV and congenital anomalies (Fig. 2, Supplementary Table S3).

Analysis of the growth trajectories showed that both CRL and EV growth trajectories were associated with SGA at birth. EV growth trajectories were also associated with congenital anomalies. No associations were found between embryonic growth trajectories and preterm birth.

### Sensitivity analyses

Analyses for adverse birth outcome in the full cohort (n = 1241), including both strictly and CRL-dated pregnancies, showed comparable associations, with odds ratios comparable to those of the main study group (Supplementary Fig. S2). Growth trajectory analyses of the full cohort were comparable to those of the main study group.

## Discussion

This periconceptional cohort study shows that, already from 7 weeks of gestation, embryonic size, as measured by 3D-US and VR, is associated with the composite outcome ‘adverse birth outcome’. Embryonic size and growth are negatively associated with SGA and birth weight, meaning that pregnancies with an embryo <20th percentile have a higher odds of delivering an SGA neonate. There was no consistent association with the other birth outcomes.

Our study builds on earlier work by ours and others (Bukowski *et al.*, 2007; Mook-Kanamori *et al.*, 2010; van Uitert *et al.*, 2013; Patel *et al.*, 2022; Xu *et al.*, 2022). By measuring embryonic size already from 7 weeks onward combined with the use of 3D-US and VR, as well as by using EV, this large prospective cohort study adds new knowledge.

The observed associations between the embryonic measures and adverse birth outcomes are mainly explained by the association between embryonic size and SGA. Almost all previous studies on this subject reported this association from around 10 weeks of gestation. We extended this period to the earlier embryonic period, with effect sizes in range with those of earlier studies (Mook-Kanamori *et al.*, 2010; van Uitert *et al.*, 2013).

This association, in this very early stage of pregnancy, supports the DOHaD paradigm that the process of becoming SGA (partly) originates in the periconceptional period (Steegers-Theunissen *et al.*, 2013). Although some individuals who are small as embryos and at birth may be constitutionally small, others might be small due to (tissue-specific) growth disturbances induced by (epi)genetic changes or direct toxic effects in the periconceptional period (Stephenson *et al.*, 2018). These changes can be the result of exposure to adverse lifestyle factors during a critical period of development, including the stages of gametogenesis, placentation, and embryogenesis (Fleming *et al.*, 2018). Previous studies have already linked early fetal size to SGA and long-term health problems (Mook-Kanamori *et al.*, 2010; Simic *et al.*, 2017; Xu *et al.*, 2022). As we have now shown that embryonic size is associated with birth outcome, long-term follow-ups should be performed to assess whether these findings in very early pregnancy translate into long-term adverse health effects (Barker, 2007; Fleming *et al.*, 2018; Stephenson *et al.*, 2018).

We were not able to reproduce the association between embryonic size and preterm birth as found by others (Mook-Kanamori *et al.*, 2010; Xu *et al.*, 2022). This may be explained by differences between study populations (general versus our tertiary hospital population), US measurement techniques, sample sizes, and statistical methods. We believe that there are multiple underlying mechanisms causing the different investigated birth outcomes variates; while SGA is often related to (epi)genetic variations or impaired placental function, common causes of preterm birth, such as intra-uterine infection, evolve later in pregnancy and may therefore not be identified by evaluating embryonic size and growth.

A previous study amongst pregnancies derived from the same source population found that embryos with a congenital anomaly had a smaller EV but not CRL (Baken *et al.*, 2017). This was no longer reproducible in our study although growth analyses showed that EV growth trajectories were associated with congenital anomalies. This is likely the result of the small number of congenital anomalies (n = 37) and the wide diversity of the included anomalies in our study.

With or without the use of 3D-US and VR, the availability of volumetric measurements of the embryo nowadays provides opportunities to more precisely measure embryonic growth. As compared to the traditionally used CRL, EV has a larger relative increment in the same period, and growth deviations may thus be detected earlier (Rousian *et al.*, 2010; Smeets *et al.*, 2012). In this study, however, we did not find strong arguments for superiority of EV above CRL in early recognition of embryos at risk of adverse birth outcomes. This might be partly explained by the larger number of missing measurements for EV due to technical or physical limitations.

From a statistical point of view, longitudinal measurements and models provide added value as they are better equipped to deal with unbalanced measurements (i.e. the 3D-US have not all been performed at the same GA) and missing data. Moreover, it may provide extra value when the relevant signal is included in the longitudinal rather than in the cross-sectional design: i.e. when slower growth rather than just being small is associated with adverse birth outcome. As such, we were surprised to find that the growth trajectory analyses added little additional value to cross-sectional measurements. This might be explained by relatively small individual changes in growth and large inter-individual growth variation in the observation period of 5 weeks. Extending growth analyses into the second and third trimesters may add information to detect pregnancies at risk, but the clinical value requires critical evaluation.this is explained by too small

As long as the exact moment of implantation of embryos is unknown in natural pregnancy, pregnancy dating will remain a subject for debate. To limit bias by the method of pregnancy dating (i.e. CRL dating versus LMP dating), we primarily studied a group of pregnancies with the most reliable estimation of pregnancy dating (i.e. natural pregnancies with regular cycle duration and LMP and those conceived via ART).

To increase the generalizability to clinical practice, where CRL dating is common practice, we additionally performed sensitivity analyses in the full cohort, including all forms of pregnancy dating. The associations found in the full cohort were comparable to those in the strictly dated group, but less pronounced. This supports our hypothesis that dating on CRL (with adjustment of estimated pregnancy duration based on embryonic size) can lead to underestimation of abnormal embryonic growth, limit the reliability of pregnancy duration, and dilute individual growth variation.

Limitations of the study include the selection of participants from a tertiary hospital setting, with many ART pregnancies (40%), reducing generalizability to general population and practice. Second, by study protocol, no 3D-US data were available in the second and third trimesters of pregnancy, which may have limited the power of the growth trajectories. In addition, not all participants had (successful) 3D-US scanning or measurements at all three time-points, due to no-show amongst other reasons and because success rates of US scanning and off-line measurements were influenced by maternal BMI and embryonic size. Last, for the used measures, we were only able to include intercorrelation coefficients, and not the absolute difference per measure (Bland–Altman plots) for interpretation of the intra- and interobserver reproducibility.

One could consider the use of in-house techniques and software a limitation, which may hamper the reproducibility by others. However, the increasing use of artificial intelligence techniques is expected to improve the external generalizability and reproducibility of 3D-measurements in the coming years. Also, by using an arbitrary cut-off of the 20th percentile for CRL and EV growth, in line with Mook-Kanamori *et al.* (2010), we assume that the risk for adverse outcome is the same in embryos growing on the first percentile as with those growing on the ninth percentile. We deem this nevertheless the best method for clinical applicability and readability.

By using multiple imputations for the covariates, we have tried to limit bias resulting from missing data. Lastly, we were not able to analyze early pregnancy loss as an outcome, which is of high clinical interest. The high rate of missing participant and fetal data in these cases were considered not at random and therefore imputation was not deemed feasible.

Strengths of the study include the cohort set-up with data collection from early pregnancy until delivery, with frequent questionnaires and serial 3D-US with EV measurements.

### Implications for clinical care and research

With this study, we aim to draw attention to the importance of the first trimester of pregnancy. We demonstrated that embryonic size and growth deviations associated with adverse birth outcome may be detected as early as from 7 weeks of gestation onward using clinically available growth measures. Preventing or limiting embryonic growth deviations may not only be relevant for birth outcome but potentially also key for health on the long-term. The DOHaD paradigm supports that early stage of pregnancy is critical for fetal, neonatal, childhood, and adult health (Barker, 2007). In future research, it would be of great interest to investigate specific subgroups, including pregnancies after oocyte donation and ART pregnancies, as well as other outcomes, including early pregnancy loss and long-term health.

The next step is to identify interventions, such as www.smarterpregnancy.co.uk, that can improve embryonic growth, and to investigate whether these interventions improve outcomes (Oostingh *et al.*, 2020; van Dijk *et al.*, 2020; van der Windt *et al.*, 2022). As compared to those initiated in later stages of pregnancy, earlier interventions may be more effective in improving embryonic, fetal, and placental health (Barker *et al.*, 2018; Stephenson *et al.*, 2018). Such interventions should primarily focus on improvements of modifiable lifestyle factors, such as folic acid and micronutrient use, quitting smoking and drinking alcohol, and a healthy dietary pattern (Twigt *et al.*, 2012; van Dijk *et al.*, 2017; Oostingh *et al.*, 2020; Hoek *et al.*, 2021).

Our data confirm previous findings in the literature, suggesting that embryonic growth is not uniform (Bottomley *et al.*, 2009; van Uitert *et al.*, 2013). This contributes to the discussion on methods used in clinical practice for pregnancy dating. In clinical practice, CRL is most often used for pregnancy dating, even in those with a known and regular menstrual cycle (Savitz *et al.*, 2002). By default, CRL-based pregnancy dating results in uniform embryonic size at the time of pregnancy dating and a missed opportunity for early identification of embryos at risk for adverse birth outcomes and opportunities to intervene.

In conclusion, this study shows that associations between embryonic size and growth and adverse birth outcomes, most specifically SGA, can already be detected from 7 weeks of gestation onward. This expands the window of opportunity to enable the development of future interventions to potentially improve pregnancy outcomes and offspring health during their life-course.

## Supplementary Material

deae212_Supplementary_Figure_S1

deae212_Supplementary_Figure_S1

deae212_Supplementary_Table_S1

deae212_Supplementary_Table_S2

deae212_Supplementary_Table_S3

## Data Availability

The data underlying this article will be shared on reasonable request to the corresponding author.
