# Supplementary figures and images for: Embryonic size and growth and adverse birth outcomes: the Rotterdam Periconception Cohort
Source: Hum Reprod. 2024 Sep 17;39(11):2434–41. doi: 10.1093/humrep/deae212 (PMC11532603; doi:10.1093/humrep/deae212)

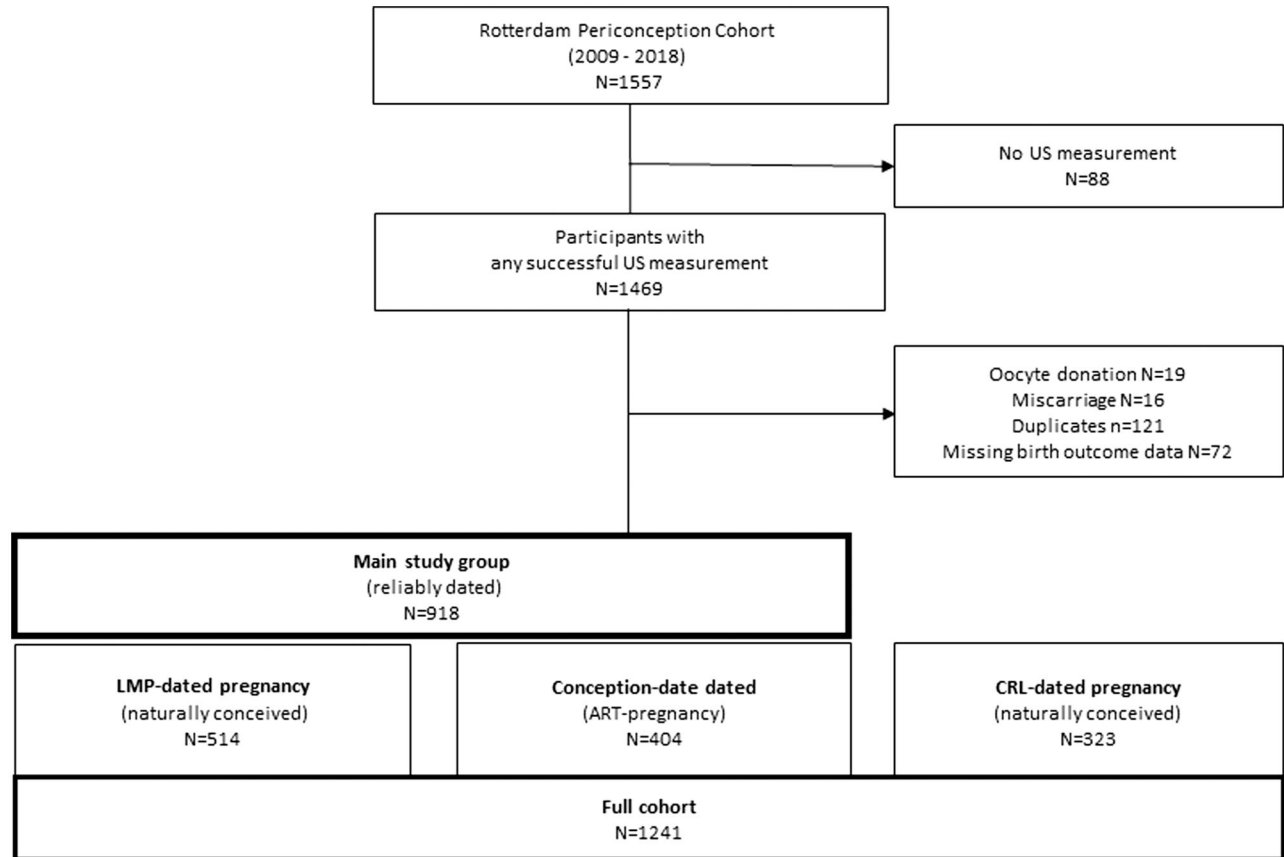

Supplementary Figure S1. Flowchart of study participants.

Supplement: deae212_Supplementary_Figure_S1 [file deae212_supplementary_figure_s1.pdf]
