# Supplementary material for: Embryonic size and growth and adverse birth outcomes: the Rotterdam Periconception Cohort
Source: Hum Reprod. 2024 Sep 17;39(11):2434–41. doi: 10.1093/humrep/deae212 (PMC11532603; doi:10.1093/humrep/deae212)
Supplement: deae212_Supplementary_Table_S1 [file deae212_supplementary_table_s1.pdf]

OXFORD
human reproduction

Human Reproduction, 2024, 1–1
https://doi.org/10.1093/humrep/deae212
Supplementary Data

Supplementary Table S1. Baseline characteristics of eligible study population.

		Eligible population (n = 1557)	Excluded (n = 316)	Included (n = 1241)	Missing data (n)
Age (years)		32.2 (29.1–35.4)	33.1 (29.4–35.9)	32.0 (29.1–35.2)	385/78/307
Geographical background	*Western*	1235 (83%)	220 (80%)	1015 (84%)	69/41/28
	Non-western	253 (17%)	55 (20%)	198 (16%)	
Educational level	*High*	825 (57%)	162 (61%)	663 (56%)	103/49/54
	Middle	505 (35%)	83 (31%)	422 (36%)	
	Low	124 (8%)	22 (8%)	102 (9%)	
Periconceptional BMI (*kg/m*^{2})		23.8 (21.4–27.3)	23.8 (21.3–28.1)	23.8 (21.4–27.0)	60/37/23
Folic acid use		1475 (99%)	270 (98%)	1205 (99%)	59/39/20
	Preconceptional initiation	1175 (81%)	210 (79%)	965 (81%)	102/51/51
Periconceptional smoking		249 (17%)	40 (14%)	209 (17%)	57/39/16
Nulliparous		784 (52%)	97 (35%)	554 (47%)	37/37/0
Conception mode	*Natural pregnancy*	995 (66%)	158 (58%)	837 (67%)	44/44/0
	ART	518 (34%)	114 (42%)	404 (33%)	

Data are presented in median (interquartile range) or number (valid percentages). Valid percentages are presented to increase comparability between groups as missing values are not equally distributed between excluded and included pregnancies.
BMI, body mass index; kg, kilograms; ART, assisted reproductive technology
