# Supplementary material for: Embryonic size and growth and adverse birth outcomes: the Rotterdam Periconception Cohort
Source: Hum Reprod. 2024 Sep 17;39(11):2434–41. doi: 10.1093/humrep/deae212 (PMC11532603; doi:10.1093/humrep/deae212)
Supplement: deae212_Supplementary_Table_S2 [file deae212_supplementary_table_s2.pdf]

OXFORD
human reproduction

Human Reproduction, 2024, 1–1
https://doi.org/10.1093/humrep/deae212
Supplementary Data

Supplementary Table S2. Baseline characteristics per method of pregnancy dating.

		Dated on LMP (N = 514)	Dated on date of conception (ART) (N = 404)	Dated on CRL (N = 323)	Missing data
Conception mode	*Natural pregnancy*	514 (100%)	NA	323 (100%)	0
	ART	NA	404 (100%)	NA	**0**
**Maternal characteristics**					
Age *(years)*		31.9 (29.0–35.2)	32.8 (29.9–36.0)	30.7 (28.1–33.7)	307
Geographical background	*Western*	430 (84%)	340 (84%)	245 (76%)	28
	Non-Western	78 (15%)	58 (14%)	62 (19%)	
Educational level	*High*	305 (59%)	205 (51%)	153 (47%)	54
	Middle	157 (31%)	153 (38%)	112 (35%)	
	Low	40 (8%)	30 (7%)	32 (10%)	
Periconceptional BMI *(kg/m^2)*		23.9 (21.6–27.1)	23.6 (20.9–26.4)	24.0 (21.5–27.8)	23
Folic acid use		502 (98%)	402 (100%)	301 (93%)	20
	Preconceptional initiation	363 (71%)	384 (95%)	218 (67%)	33
Periconceptional smoking		90 (18%)	52 (13%)	67 (21%)	16
Nulliparous		232 (45%)	308 (76%)	147 (46%)	0
**Neonatal characteristics**					
Gestational age at birth *($weeks^{+days}$)*		39^{+0} (38^{+0}–40^{+1})	39^{+3} (38^{+2}–40^{+2})	39^{+1} (37^{+6}–40^{+1})	0
	Preterm birth	46 (9%)	27 (7%)	38 (12%)	0
Birth weight *(gram)*		3332 (2993– 3701)	3350 (3005– 3660)	3270 (2913–3658)	0
Birth weight percentile		48 (24–73)	39 (20–70)	45 (20–74)	0
	SGA	61 (12%)	49 (12%)	48 (15%)	0
Sex	*Male*	261 (51%)	109 (51%)	158 (50%)	0
Major congenital anomaly		13 (3%)	16 (4%)	8 (3%)	43
Mortality	*Fetal*	4 (<1%)	7 (2%)	6 (2%)	0
	Early neonatal	2 (<1%)	2 (<1%)	0 (0%)	0
Adverse birth outcome		107 (21%)	92 (23%)	88 (27%)	0

Data are presented in median (interquartile range) or number (percentages).
LMP, last menstrual period; SGA, small for gestational age, NA; not applicable.
