# Supplementary material for: Embryonic size and growth and adverse birth outcomes: the Rotterdam Periconception Cohort
Source: Hum Reprod. 2024 Sep 17;39(11):2434–41. doi: 10.1093/humrep/deae212 (PMC11532603; doi:10.1093/humrep/deae212)
Supplement: deae212_Supplementary_Table_S3 [file deae212_supplementary_table_s3.pdf]

OXFORD human reproduction

Human Reproduction, 2024, 1–1
https://doi.org/10.1093/humrep/deae212
Supplementary Data

Supplementary Table S3. Associations of embryonic size <20th percentile and adverse birth outcome in the main study group (n = 918).

			Model 1			Model 2		
**Adverse birth outcome**		**N**	**OR**	**95% CI**	***P* value**	**OR**	**95% CI**	***P* value**
**7 weeks**								
CRL	*(mm)*	479	2.14	1.30–3.52	**0.003**	2.03	1.21–3.39	**0.007**
EV	*(cm^3)*	412	1.70	0.96–3.01	0.07	1.66	0.91–3.01	0.16
**9 weeks**								
CRL	*(mm)*	698	1.16	0.72–1.87	0.55	1.21	0.73–1.98	0.46
EV	*(cm^3)*	617	1.46	0.86–2.48	0.16	1.49	0.86–2.57	0.16
**11 weeks**								
CRL	*(mm)*	754	1.38	0.91–2.09	0.13	1.42	0.92–2.18	0.11
EV	*(cm^3)*	586	1.52	0.95–2.44	0.08	1.48	0.91– 2.39	0.11
Preterm birth								
**7 weeks**								
CRL	*(mm)*	417	1.81	0.84–3.90	0.13	1.59	0.73–3.42	0.24
EV	*(cm^3)*	358	2.29	1.00–5.24	**0.05**	2.04	0.88–4.71	0.09
**9 weeks**								
CRL	*(mm)*	604	1.07	0.49–2.32	0.87	1.06	0.49–2.30	0.89
EV	*(cm^3)*	532	1.45	0.63–3.34	0.39	1.35	0.59–3.13	0.48
**11 weeks**								
CRL	*(mm)*	656	0.98	0.50–1.92	0.95	0.96	0.49–1.88	0.90
EV	*(cm^3)*	506	1.42	0.70–2.88	0.33	1.41	0.69–2.85	0.34
SGA								
**7 weeks**								
CRL	*(mm)*	437	2.37	1.29–4.38	**0.005**	2.18	1.16–4.09	**0.02**
EV	*(cm^3)*	377	1.69	0.83–3.44	0.14	1.66	0.79–3.50	0.18
**9 weeks**								
CRL	*(mm)*	597	1.48	0.84–2.61	0.17	1.59	0.88–2.86	0.13
EV	*(cm^3)*	524	2.00	1.09–3.69	**0.03**	2.09	1.10–3.97	**0.03**
**11 weeks**								
CRL	*(mm)*	647	2.00	1.24–3.25	**0.005**	2.10	1.27–3.50	**0.004**
EV	*(cm^3)*	506	2.19	1.29–3.71	**0.004**	2.17	1.25–3.78	**0.006**
CONGENITAL ANOMALIES								
**7 wee**k								
CRL	*(mm)*	377	2.69	0.90–7.99	0.07	2.73	0.96–7.74	0.06
EV	*(cm^3)*	328	1.60	0.46–5.60	0.73	1.58	0.48–5.21	0.46
**9 weeks**								
CRL	*(mm)*	555	0.98	0.30–3.14	0.97	0.94	0.30–2.92	0.92
EV	*(cm^3)*	486	0.97	0.25–3.76	0.96	0.95	0.26–3.49	0.94
**11 weeks**								
CRL	*(mm)*	592	0.97	0.30–3.16	0.96	1.03	0.34–3.17	0.92
EV	*(cm^3)*	456	0.99	0.25–3.96	0.99	1.00	0.28–3.63	0.96

Statistically significant findings ($P < 0.05$) are in **bold**.
Adverse outcomes include: combined outcome of preterm birth, small for gestational age, mortality, and congenital anomalies.
Model 1: adjusted for GA at US measurement.
Model 2: adjustment for Model 1 + educational level (high/not-high), maternal smoking, and fetal sex, parity (nulliparous/multiparous), maternal age, maternal periconceptional BMI, maternal geographical background (Western/non-Western), and mode of conception (spontaneous/ART).
n, number; OR, odds ratio; CRL, crown–rump length; EV, embryonic volume; mm, millimeter; cm^3, cubic centimeter; GA, gestational age; US, ultrasound.

© The Author(s) 2024. Published by Oxford University Press on behalf of European Society of Human Reproduction and Embryology.
This is an Open Access article distributed under the terms of the Creative Commons Attribution License (https://creativecommons.org/licenses/by/4.0/), which permits unrestricted reuse, distribution, and reproduction in any medium, provided the original work is properly cited.
Human Reproduction, 2024, 00, 1–13
https://doi.org/10.1093/humrep/deae212
Original Article
